# Quantitative analysis of Hedgehog gradient formation using an inducible expression system

**DOI:** 10.1186/1471-213X-7-43

**Published:** 2007-05-07

**Authors:** Vivian F Su, Kelly A Jones, Michael Brodsky, Inge The

**Affiliations:** 1 Program in Gene Function and Expression, Department of Molecular Medicine, University of Massachusetts Medical School, Worcester, MA, USA; 2 Cancer Research Center, University of Hawaii at Manoa, Honolulu, HI, USA; 3 Department of Developmental Biology, Utrecht University, Utrecht, the Netherlands

## Abstract

**Background:**

The Hedgehog (Hh) family of secreted growth factors are morphogens that act in development to direct growth and patterning. Mutations in human Hh and other Hh pathway components have been linked to human diseases. Analysis of Hh distribution during development indicates that cholesterol modification and receptor mediated endocytosis affect the range of Hh signaling and the cellular localization of Hh.

**Results:**

We have used an inducible, cell type-specific expression system to characterize the three-dimensional distribution of newly synthesized, GFP-tagged Hh in the developing *Drosophila *wing. Following induction of Hh-GFP expression in posterior producing cells, punctate structures containing Hh-GFP were observed in the anterior target cells. The distance of these particles from the expressing cells was quantified to determine the shape of the Hh gradient at different time points following induction. The majority of cholesterol-modified Hh-GFP was found associated with cells near the anterior/posterior (A/P) boundary, which express high levels of Hh target genes. Without cholesterol, the Hh gradient was flatter, with a lower percentage of particles near the source and a greater maximum distance. Inhibition of Dynamin-dependent endocytosis blocked formation of intracellular Hh particles, but did not prevent movement of newly synthesized Hh to the apical or basolateral surfaces of target cells. In the absence of both cholesterol and endocytosis, Hh particles accumulated in the extracellular space. Staining for the Hh receptor Ptc revealed four categories of Hh particles: cytoplasmic with and without Ptc, and cell surface with and without Ptc. Interestingly, mainly cholesterol-modified Hh is detected in the cytoplasmic particles lacking Ptc.

**Conclusion:**

We have developed a system to quantitatively analyze Hh distribution during gradient formation. We directly demonstrate that inhibition of Dynamin-dependent endocytosis is not required for movement of Hh across target cells, indicating that transcytosis is not required for Hh gradient formation. The localization of Hh in these cells suggests that Hh normally moves across both apical and basolateral regions of the target cells. We also conclude that cholesterol modification is required for formation of a specific subset of Hh particles that are both cytoplasmic and not associated with the receptor Ptc.

## Background

Members of the Hh family have an evolutionarily conserved role in regulating growth and differentiation during development of many organisms [1]. Hh directly induces different cell fates in a concentration dependent manner, and thus is classified as a morphogen. This concentration gradient is tightly regulated and any disruption can cause abnormal cell specification [2,3]. Mutations in Hh pathway components have also been shown to lead to human disorders and disease [4].

In *Drosophila*, the Hh morphogen is produced and secreted from posterior compartment cells in embryos and imaginal wing discs. Hh travels to anterior target cells and forms a concentration gradient from its source [1,5]. Models that have been proposed for morphogen distribution and gradient formation include extracellular movement by diffusion and intracellular movement by transcytosis [4,5]. The diffusion model can be further divided into the free diffusion and restricted planar diffusion models. In the free diffusion model, the morphogen is secreted from the producing cells into the extracellular space and diffuses in three-dimensions out to the target cells. In the restricted planar diffusion model, the morphogen moves directly from cell to cell, always remaining in the two-dimensional epithelial cell layer close to the cell surface. In contrast, the transcytosis model proposes a unique mechanism where successive rounds of endocytosis and exocytosis move Hh through cells [4,5].

Hh proteins in all organisms are dually lipid modified as part of their intracellular processing to produce HhNp (p for processed) and these modifications are likely to affect movement of the morphogen. In *Drosophila*, Hh is synthesized as a 45 kDa full length precursor protein that undergoes an autoproteolytic cleavage [6,7]. Cholesterol is covalently attached to the C-terminus of the N-terminal signaling molecule as part of this process [8]. A palmitoyl group is attached at the N-terminus by a membrane bound O-acyltransferase to produce a dually lipidated 19 kDa HhNp molecule [9-13]. Because the protein is lipid modified, Hh movement must include a mechanism that prevents this modification from restricting Hh to the membranes of the producing cells. One mechanism to mobilize lipid modified Hh may be to form micelle-like structures; in gel filtration assays, Hh and vertebrate Sonic Hedgehog (Shh) multimers, which require both lipid modifications, can be detected [14,15]. This high molecular weight Hh fraction will associate with cell membranes in tissue culture cells while the monomeric forms do not [16,17]. The hydrophobic moieties could be hidden inside multimeric micelle-like structures to make the HhNp complexes more soluble in order to diffuse [15,18]. Therefore, the cholesterol modification could be required for multimerization that enables long range movement and proper gradient formation. The requirement for cholesterol modification in signaling is not clear due to conflicting reports from both *Drosophila *and mammalian studies. In some studies, using *Drosophila *wing discs and mouse limb buds, the unmodified Hh (HhN) has long range activity [8,14,19-21]. However, other *Drosophila *and mammalian studies suggest that cholesterol is required for long range activity [16,22-24].

In *Drosophila *and vertebrates, the extracellular matrix components Heparan Sulfate Proteoglycans (HSPGs) are involved in Hh movement. Loss of HSPGs block Hh movement and signaling in adjacent wild-type cells is impaired [25-31]. Interestingly, HSPG regulation of Hh movement depends on cholesterol, as unmodified HhN is unaffected by the loss of HSPGs [14]. The cholesterol may mediate Hh and HSPG association, as cholesterol-modified Hh and Shh are able to bind heparin [6,32]. One interpretation of these results is that HSPGs are required to mediate planar diffusion of cholesterol-modified Hh across or through target cells, but that unmodified Hh is able to move via free diffusion. In support of this model, unmodified Hh expressed in the peripodial cell layer of the developing *Drosophila *wing can move through the extracellular space of the wing lumen while cholesterol-modified Hh is restricted to the layer of cells in which it is expressed [14,22].

Receptor-mediated endocytosis has also been proposed to regulate the spreading of Hh. In addition to transducing the Hh signal, Patched (Ptc), the receptor for Hh, has been shown to sequester and limit the range of distribution by binding and internalizing Hh [33,34]. Hh is thought to be primarily endocytosed together with Ptc and then targeted for degradation [14], although Ptc-independent cytoplasmic Hh particles have been detected as well [34,35]. A role for endocytosis in Hh gradient formation has been proposed, either as part of transcytosis or by removing Hh to limit the distribution range. Blocking endocytosis in embryos and wing discs with a *dynamin *mutant (*shibire *in *Drosophila *or *shi*) does not appear to affect Hh target gene expression or spreading [14,16,28,34]. These observations suggest that Dynamin-mediated endocytosis may be required for Hh degradation but not Hh distribution. However, in these experiments, *shi *is inactivated in tissues with a preexisting gradient of Hh. It is unclear whether the Hh distribution observed reflects this preexisting pool or newly synthesized Hh produced following *shi *inactivation.

While previous studies provided much information about Hh distribution, these studies also have some limitations that may contribute to conflicting conclusions. One limitation of previous studies was that overexpressed Hh and/or preexisting pools of Hh, instead of newly produced protein, were examined [36]. Therefore, the observed distribution of Hh may reflect redistribution of preexisting protein instead of the ability of Hh to move despite the endocytosis block. This issue can be addressed by using an inducible system where the movement of newly synthesized protein is studied. Another limitation is that Hh distribution was examined after the Hh gradient had reached a steady state; analysis during gradient formation may provide more information about Hh movement. This too can be addressed with an inducible system. Finally, the ability to determine the magnitude and statistical significance of changes in Hh distribution may be limited by the potential difficulty in measuring the three-dimensional distance traveled from the producing cells.

We have attempted to resolve these issues by quantitatively investigating the distribution of GFP-tagged forms of Hh protein using an inducible expression system. To determine whether the cholesterol modification and/or endocytosis have any effect on the process of gradient formation, we expressed functional Hh-GFP with and without the cholesterol-modification in a wild-type or *shi*^*ts*1 ^mutant background. Specifically, the Gal80-Gal4 system was used to temporally express (pulse) Hh-GFP transgenes in their normal expression domain (the posterior compartment of the developing wing); this method allows the rate of Hh gradient formation over time to be observed. We demonstrate that both HhNp-GFP and HhN-GFP are present in punctate structures (particles). We have also developed a system for quantitative measurement of Hh distribution and found that HhN-GFP migrates further than HhNp-GFP while less is retained near the expressing cells. We demonstrate that when endocytosis is blocked, newly synthesized HhNp-GFP is still detected in particles and can still move anteriorly, arguing against an essential role of transcytosis. Lumenal HhN-GFP particles were observed, indicating cholesterol is responsible for retaining Hh on cell surfaces. We propose that HhNp and HhN spread through both apical and basolateral regions by planar diffusion and that the cholesterol modification serves to retain Hh on the cell surface and promotes the formation of a steep gradient.

## Results

### Generation of Hh-GFP fusion constructs and functional characterizations

**Figure 1:**
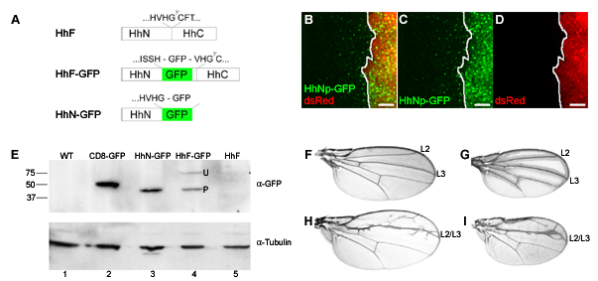
**HhNp-GFP is functional**. (A) Scheme of HhF-GFP (middle) and HhN-GFP (bottom) fusion constructs and predicted processing as compared to wild-type HhF (top). HhF-GFP is predicted to be processed into HhNp-GFP. (B-D) HhNp-GFP (green, C) is expressed in posterior cells labeled by fluorescent protein dsRed (red, D) and secreted (B), similar to wild-type HhNp (A/P boundary marked by a solid white line). Scale bar: 5 μm (E) Western blot of salivary gland protein extracts labeled with anti-GFP (upper panel) or tubulin (lower panel). Upper panel: as negative controls we used extracts of wild-type w^1118 ^larvae (lane 1) and larvae expressing an untagged HhF (lane 5), CD8-GFP was used as a positive control (lane2). A single 46 kDa band is seen in the lane with HhN-GFP expressing larvae (lane 3) and in the lane loaded with extract of HhF-GFP expressing larvae, two bands of 70 kDa and 46 kDa are seen (lane 4; U: unprocessed full-length HhF-GFP, P: processed HhNp-GFP). Lower panel: the same blot was reprobed with anti-tubulin for loading control. (F-I) HhF expression in adult wings. (F) Wild-type wing. (G) Wing from HhF-GFP rescue of *hh*^*GS*1 ^mutant has a similar phenotype to wild-type. (H) As a positive control, untagged HhF is expressed with 71B-Gal4 resulting in merged veins L2 and L3. (I) Ectopic expression of HhF-GFP has a similar phenotype to untagged HhF.

To study movement and distribution of newly synthesized Hh, we generated Hh-GFP fusion proteins that are suitable for both live and fixed tissue experiments. The HhF-GFP fusion construct (Figure 1A) contains GFP coding sequences placed between Hh amino acids 254 (H) and 255 (V). The same location was used previously to generate functionally tagged HhNp [14,19,34], which is expected to be processed into a HhNp-GFP and an untagged Hh-C. To express unprocessed HhN-GFP, we generated an expression construct that encodes the N-terminus of Hh fused to GFP, similar to a HhN-GFP fusion described previously [37].

Several experiments were performed to determine whether HhNp-GFP is functional. First, HhNp-GFP was constitutively expressed with the Gal4-UAS system [38] using Hh-Gal4, which would express the transgene using the endogenous *hh *promoter. DsRed protein was co-expressed with the Hh fusion protein to identify expressing cells and mark the anterior/posterior (A/P) boundary in this and subsequent experiments. HhNp-GFP was secreted from the dsRed-expressing cells similar to untagged wild-type HhNp (Figure 1B–D). Second, HhNp-GFP and HhN-GFP were expressed in salivary glands using the ubiquitous 71B-Gal4 driver and the glands were extracted to identify the Hh fusion proteins on a Western blot (Figure 1E). For HhN-GFP, an approximately 46 kDa protein band was detected corresponding to the predicted size of the N-terminus fused to GFP (lane 3). For HhNp, an approximately 70 kDa band representing the uncleaved full length HhF-GFP protein (U) and a 46 kDa band representing the cleaved HhNp-GFP signaling molecule (P, lane 4) were observed. These results indicate that both Hh-GFP fusion proteins are expressed and properly processed. Third, we tested the ability of HhNp-GFP to rescue the embryonic lethality of homozygous *hh*^*GS*1 ^mutants. Expressing HhNp-GFP in the posterior compartment using the En-Gal4 driver, we were able to fully rescue *hh*^*GS*1 ^mutants to adulthood. These animals appeared to develop normally, as demonstrated by a normal though slightly smaller wing (Figure 1G). Finally, wings of flies ectopically expressing functional untagged HhNp [7] and wings expressing HhNp-GFP had similar phenotypes of merged wing veins L2 and L3 (Figure 1H and 1I). These results demonstrate that the Hh-GFP fusion proteins are properly synthesized, and that HhNp-GFP has the same properties as previously described for wild-type HhNp [39].

### Analysis of HhNp-GFP localization in living tissue

**Figure 2:**
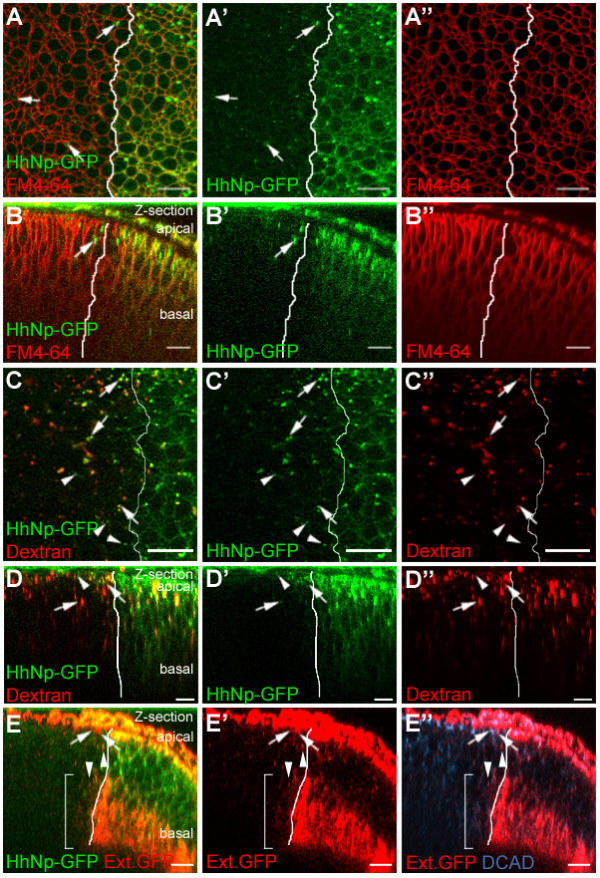
**HhNp-GFP localizes at the membrane, in endocytic compartments, and extracellularly**. (A-E) Localization of HhNp-GFP (green), expressed with Hh-Gal4, with FM4-64 (red, A-B), dextran (red, C-D), extracellular labeling with anti-GFP, and DCAD (red and blue, E). (B, D, E) Z-sections. HhNp-GFP (A') co-localizes with FM4-64 (A") at the membrane in the posterior (A/P boundary marked by the solid white line) as seen in the merge (A). Anterior HhNp-GFP appears in particles (arrows). Most of the particles localize apically (B). Many of the anterior HhNp-GFP particles co-localize (arrows in C-D) with dextran but some can be found without dextran (arrowheads in C-D). Incubation of anti-GFP in cold medium detects extracellular HhNp-GFP in the anterior apically in particles (arrows in E), and basolaterally both in particles (arrowheads in E) and with a membrane association (bracket in E). Scale bar: 8 μm

The *Drosophila *larval wing imaginal disc consists of two layers of epithelial cells, separated by the peripodial lumen. On the apical side, a squamous epithelial layer forms the peripodial membrane, while on the basal side, the disc epithelium is found that will give rise to the wing and notum. Most previous studies exploring HhNp localization in the disc epithelium have been performed with fixed discs. In these studies and our own experiments with fixed discs, most HhNp in the anterior compartment is found in punctate structures, as detected by immunostaining. To rule out the possibility of fixation-induced alterations in Hh localization, we took advantage of the GFP tag to examine the localization of HhNp-GFP in live tissues. Hh-Gal4 was used to express HhNp-GFP in the endogenous Hh expression domain in the posterior compartment of the wing imaginal disc [40]. In live discs, HhNp-GFP co-localized with the membrane marker FM4-64 in the posterior compartment and was also found in particles in both the posterior and anterior compartments (Figure 2A). These particles were found in both apical and basolateral regions (Figure 2B). HhNp has previously been observed in endosomes [14,16,34,35]. We used endocytosed dextran to determine whether the particles containing HhNp-GFP in live discs included endosomes. In both the anterior and posterior compartments, many, but not all, of the HhNp-GFP particles co-localized with dextran (Figure 2C and 2D), confirming that at least some HhNp particles correspond to endosomes. The non-co-localizing HhNp-GFP vesicles could represent endosomes formed before or after dextran incubation, non-endocytic vesicles, or extracellular particles.

We then investigated the extracellular distribution of HhNp-GFP, using an "*in vivo*" extracellular labeling method as described by Strigini and Cohen [41]. In this procedure, live discs are incubated with anti-GFP antibody and then washed prior to fixation and detergent treatment. In control experiments using this method, we detected a protein with an extracellular GFP tag (GFP-Dally-like) while an intracellular YFP tag (Ptc-YFP) was not detected, demonstrating that the staining procedure reliably distinguished between extracellular and intracellular localization (Additional File 1). When similar experiments were performed for HhNp-GFP, strong extracellular staining was observed in the apical and basal regions in the posterior compartment of the disc where Hh is produced (Figure 2E). In the anterior compartment, extracellular HhNp-GFP was detected in apical particles (Figure 2E, arrows), basolateral particles (Figure 2E, arrowheads) and on the basolateral membrane (Figure 2E, bracket). Extracellular HhNp-GFP was also detected in the overlying peripodial membrane. While some of these cells align with the anterior compartment of the disc proper cells, this region corresponds to the posterior compartment of the peripodial membrane where HhNp-GFP is being expressed [42]. These localization results are consistent with previously published data for both untagged and tagged HhNp [14,22,34]. In conclusion, the largely punctate localization of tagged and native Hh observed in the anterior compartment of the developing wing after fixation accurately reflects the localization of Hh in living tissues.

### Induction and quantitative analysis of a Hh gradient

**Figure 3:**
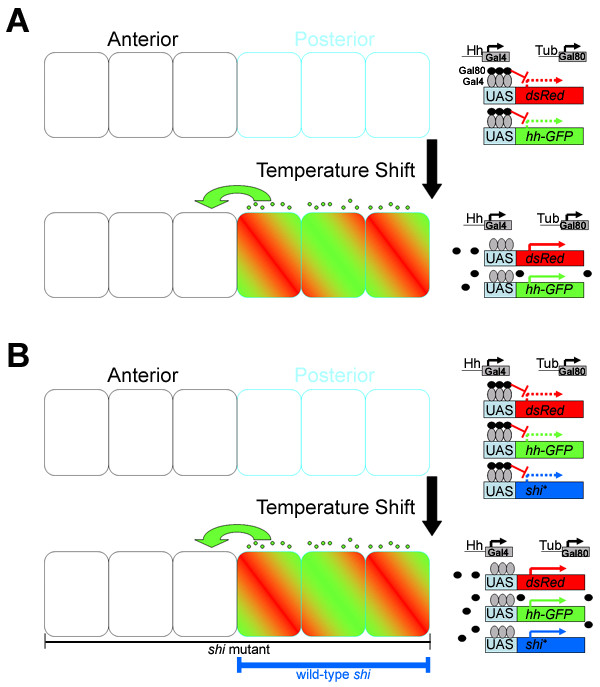
**Schematic diagram of Gal4-Gal80 inducible expression system**. (A) Inducing Hh-GFP expression. Vials are kept at 18°C, the Gal80 permissive temperature where tubulin-Gal80 blocks Gal4-mediated transcription. Upon a shift to 32°C, Gal80 repression is relieved and Gal4 transcription proceeds. Hh-GFP is expressed in the posterior cells with Hh-Gal4, and UAS-dsRed marks the expressing cells. (B) Inducing Hh-GFP expression in *shi*^*ts*1 ^mutant background. Vials are kept at 18°C, the Gal80 permissive temperature and *shi*^*ts*1 ^mutant restrictive temperature. Upon a shift to 32°C, Gal4 transcription proceeds while endocytosis is blocked. Wild-type Shi is expressed in the posterior to restore endocytosis in the expressing cells. The resulting Hh-GFP movement into the anterior would be due solely to *shi *independent mechanisms. Hh-Gal4 is used again to drive transgene expression.

**Figure 4:**
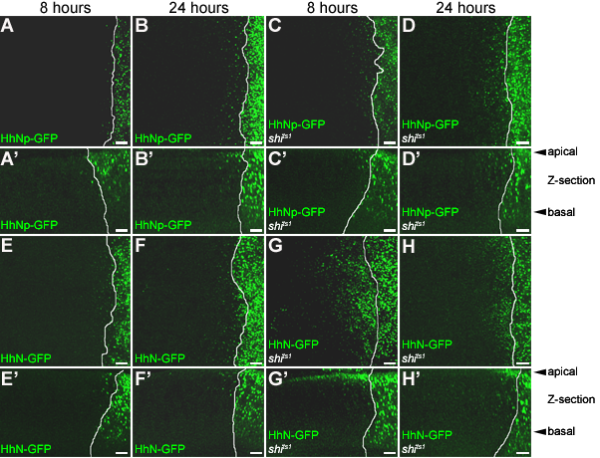
**Cholesterol restricts HhNp-GFP distribution but endocytosis is not required for distribution**. (A-H) Induced expression of HhNp-GFP in wild-type (A-B) and *shi*^*ts*1 ^background (C-D) and HhN-GFP in wild-type (E-F) and *shi*^*ts*1 ^background (G-H). (A-H) 25 μm projections; (A'-H') 20 μm Z-section projections. At 8 hr, HhNp-GFP particles are found near the A/P boundary, marked by the solid white line (A, A'). After 24 hr, more particles can be found further away (B, B'). HhN-GFP particles are detected further from the A/P boundary than HhNp-GFP at both time points (E-F). When endocytosis is blocked, HhNp-GFP particles are still detected in anterior cells (C-D). In wild-type and *shi*^*ts*1 ^backgrounds, HhNp-GFP particles appear closer to the apical side (A'-D') as well as HhN-GFP in wild-type. When endocytosis is blocked, HhN-GFP moves into the anterior but there is reduced punctate staining and more membrane accumulation (G-H), primarily on the apical side of cells (G'-H'). Scale bar: 5 μm

**Figure 5:**
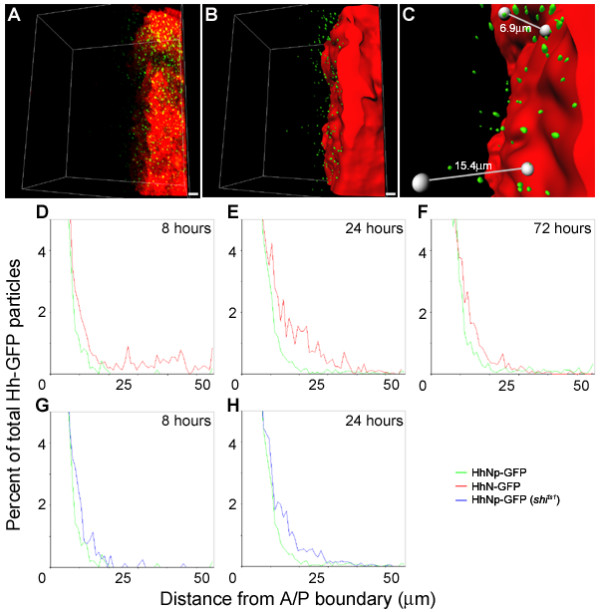
**Quantitative analysis of Hh-GFP distribution: Cholesterol is required to restrict distribution but endocytosis is not required for distribution**. (A-C) Schematic illustration of quantitative analysis. (A) Three dimensional reconstruction of a confocal z-stack with Hh-GFP (green) and dsRed (red) marking the expressing cells. (B) Generation of isosurfaces. DsRed isosurfacing was used to generate a distance map used to measure distances of Hh-GFP particles. Hh-GFP particles were isosurfaced to identify particles using an intensity threshold and size criteria. (C) Depiction of particle distance measurements. Particles were measured for the shortest distance to the expressing cells (lines depict manual measurements but all measurements were calculated in an automated fashion). Scale bar: 5 μm. (D-F) Mean of normalized HhNp-GFP (green) versus HhN-GFP (red) distribution profiles in a wild-type background. All samples were normalized to generate percentages of particles at the distances. Normalized data was then averaged to generate distribution profiles. Enlargement of the distribution near the x-axis shows more HhN-GFP is detected further from the A/P boundary (0 on the x-axis) at 8 (D; HhNp-GFP n = 5, HhN-GFP n = 4) and 24 hr (E; HhNp-GFP n = 16, HhN-GFP n = 7). The same is seen at 72 hr (F; HhNp-GFP n = 5, HhN-GFP n = 6). (G-H) Mean of normalized HhNp-GFP distribution profiles in wild-type background (green) versus *shi*^*ts*1 ^mutant background (blue). At 8 (G; *shi*^*ts*1 ^n = 4) and 24 hr (H; *shi*^*ts*1 ^n = 7), HhNp-GFP in the mutant background is less restricted and found further away from the A/P boundary than in the wild-type background. The same HhNp-GFP distribution profiles in the wild-type background from D and E are used for G and H, respectively.

In the experiments described above and by other groups, Hh distribution in the developing wing is characterized following expression over a period of several days. To examine the movement and distribution of newly synthesized Hh, we used the Gal80-Gal4 temperature-sensitive inducible system [43]. At a lower temperature, Gal80 inhibits Gal4 activity (Figure 3A). Following a shift to higher temperature, Gal80 is inactivated and Hh-Gal4 activates expression of Hh-GFP in posterior wing cells. With this system, we were able to analyze Hh distribution at different time points following induction (Figure 4). At 8 hours following induction, Hh-GFP exists as particles in the anterior compartment, primarily near the A/P boundary. At 24 and 72 hours following induction, increased numbers of particles are observed, both near the A/P boundary and further from the expressing cells (Figures 4, 5, and Additional File 2).

To examine how different factors alter the distribution of newly synthesized Hh, we developed an assay to quantify the distance of individual particles of Hh-GFP from the A/P boundary. First, a series of optical sections were transformed into a three-dimensional reconstruction of Hh-GFP and dsRed localization in a region of the wing disc near the A/P boundary (Figure 5A). Next, the dsRed expressing cells were converted into a single isosurface and the distance of Hh-GFP particles from this surface was determined (see Methods and Figure 3A–C, individual data sets are shown in Additional File 3). Because the number of particles induced was variable, the percentages of Hh-GFP particles at different distances from the A/P boundary were used to normalize distributions profiles within and between different experimental conditions (Figure 5, Additional File 4).

To establish how long the Hh gradient takes to form (Figure 4 and 5), we analyzed the change in HhNp-GFP distribution at 8, 24, and 72 hours following induction (Figure 5D–F, Table 1). At each time point, the majority of HhNp-GFP was detected within 8 μm of the A/P boundary. This distance represents the average width of the region expressing high levels of the Hh target gene *ptc *(unpublished results). The percentage of HhNp-GFP particles in this region significantly decreased from 8 to 24 hours, but not from 24 to 72 hours (Table 2). Similarly, both the median and the 90^th ^percentile values for the HhNp-GFP distributions significantly increased from 8 to 24 hours, but not from 24 to 72 hours. From this analysis, we conclude that the HhNp-GFP gradient is still forming at 8 hours and is approaching its final shape by 24 hours.

**Table 1:** Analysis of Hh-GFP particle distribution in wing discs

Time point	Sample	Median	90^th ^percentile distance	% within 8 μm
8 hr	HhNp-GFP	2.5 μm ± 0.6 μm	8 μm ± 1.4 μm	93 ± 3.2
	HhN-GFP	4.4 μm ± 3.0 μm	27 μm ± 15.8 μm	75 ± 18.4
	HhNp-GFP(*shi*^*ts*1^)	2.7 μm ± 0.4 μm	10 μm ± 1.8 μm	85 ± 6.4
				
24 hr	HhNp-GFP	3.5 μm ± 0.9 μm	11 μm ± 2.4 μm	82 ± 7.7
	HhN-GFP	6.1 μm ± 1.9 μm	25 μm ± 7.0 μm	64 ± 10.7
	HhNp-GFP(*shi*^*ts*1^)	4.8 μm ± 1.2 μm	16 μm ± 4.8 μm	70 ± 8.8
				
72 hr	HhNp-GFP	3.8 μm ± 0.6 μm	12 μm ± 2.1 μm	79 ± 8.4
	HhN-GFP	5.1 μm ± 1.2 μm	31 μm ± 3.7 μm	70 ± 11.5

Between 4–16 discs were counted for each genotype and time point. Standard deviation was calculated for each measurement.

**Table 2:** ANOVA calculated P-values for significance

	Median	90^th ^percentile distance	% within 8 um
HhNp vs HhN	<0.0001	<0.0001	<0.0001
HhNp vs HhNp(*shi*^*ts*1^)	0.1887	0.0131	0.0417
			
8 hr vs 24 hr	0.0009	0.0011	0.0131
24 hr vs 72 hr	0.8641	0.1341	0.7693

There was no significant interaction between the time factor and the genotype factor. Therefore, the significance of the main effects (time irrespective of genotype or genotype irrespective of time) are reported. P-values less than 0.05 are considered significant.

### Cholesterol modification is required for proper Hh distribution

Having identified time points when newly synthesized HhNp is forming a gradient (8 and 24 hours) or has reached a steady state (72 hours), we examined the distribution of HhN-GFP, which lacks the cholesterol modification, at these same time points. Similar to HhNp-GFP, the majority of HhN-GFP was detected within the first 8 μm from the A/P boundary (Table 1) and the median value, 90^th ^percentile, and percent of particles at 8 μm changed from 8 to 24 hours, but not from 24 to 72 hours (Table 2). However, the shape of the gradient is different for HhN than HhNp. Comparisons of composite distribution profiles reveal that the distribution of HhN-GFP is shifted further from the A/P boundary at all time points (Figures 5E–F). The median and 90^th ^percentile values are statistically significantly greater for HhN-GFP than for HhNp-GFP (Tables 1 and 2) indicating that HhN is able to move further from the producing cells. In addition, the percentage of HhN-GFP within 8 μm was significantly lower (Tables 1 and 2). This quantitative analysis extends previous studies indicating that HhN is able to move further into the anterior compartment than HhNp [14,19,20]. This difference is not simply a result of greater amounts of HhN being secreted from producing cells. Rather, the cholesterol modification of Hh contributes to the shape of the gradient. Specifically, cholesterol is required to create a steeper Hh gradient with a higher percentage near the A/P boundary and a decreased maximum distance traveled.

### Movement of newly synthesized HhNp-GFP particles does not require Shi

Several studies have shown that Hh is internalized with Ptc and localizes in endocytic compartments through a mechanism that requires the *Drosophila *Dynamin homolog Shi [14,28,34]; transient inhibition of Shi-dependent endocytosis blocks Hh internalization, but does not affect Hh-dependent gene expression. These experiments may indicate that endocytosis is not required for movement of Hh into its target cells. However, because Hh is synthesized prior to Shi inhibition, it is not clear whether the Hh observed in target cells is newly synthesized or was present prior to *shi *inactivation.

**Figure 6:**
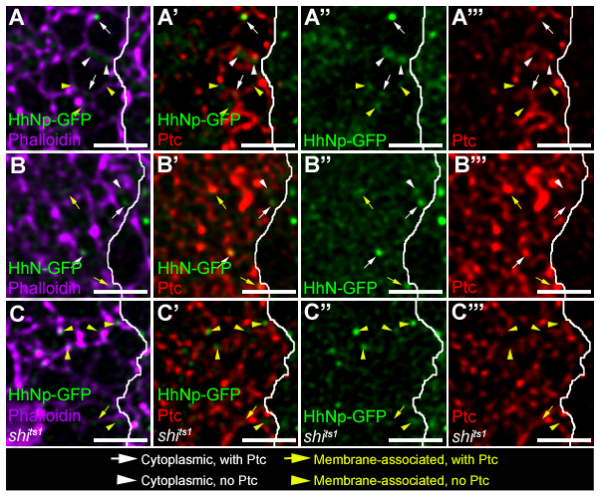
**Non-Ptc containing Hh-GFP particles require cholesterol but not endocytosis**. (A-C) Ptc co-localization with HhNp-GFP (A), HhN-GFP (B), and HhNp-GFP in the *shi*^*ts*1 ^background (C) after expression induced for 8 hr. (A-C) Hh-GFP (green) labeled with Phalloidin (purple). (A'-C') Hh-GFP (green) labeled with Ptc (red). (A''-C'') Hh-GFP only. (A'''-C''') Ptc only. 4 classes of HhNp-GFP particles are seen: non-Phalloidin associated (cytoplasmic) with Ptc (white arrow), non-Phalloidin associated (cytoplasmic) without Ptc (white arrowhead), Phalloidin (membrane) associated with Ptc (yellow arrow), Phalloidin (membrane) associated without Ptc (yellow arrowhead). Most HhNp-GFP particles are membrane-associated and do not contain Ptc, but cytoplasmic particles have a relatively even distribution with and without Ptc. More HhN-GFP also localizes with Phalloidin, and almost all cytoplasmic HhN-GFP particles contain Ptc. HhNp-GFP particles in *shi*^*ts*1 ^mutant background are Phalloidin-associated and many do not contain Ptc. The A/P boundary is marked by a solid white line. Scale bar: 5 μm.

To address this question, we used the inducible Hh-GFP system to simultaneously initiate a pulse of Hh-GFP expression in the posterior compartment and inhibit Shi function in the anterior compartment. Initially, we expressed HhNp-GFP throughout development (i.e. without Gal80) and then blocked endocytosis for 8 hours with the temperature sensitive mutation *shi*^*ts*1^; under these conditions, HhNp-GFP accumulated at the basal membranes of the anterior cells similar to previously published results [14,28,34] and in more apical punctate structures (Additional File 5). Next, the effects of simultaneously inducing Hh-GFP expression while transiently blocking endocytosis were examined. In these experiments using the Gal80-Gal4 system, the same temperature shift induces Hh-GFP and dsRed expression and inactivates *shi*^*ts*1^. In addition, wild-type Shi was also expressed in the posterior compartment, rescuing the endocytosis defect in the expressing cells (Figure 3B). Thus, Hh-GFP was induced in the posterior cells concurrently with a block in Shi-dependent endocytosis. Newly synthesized HhNp-GFP was observed in the anterior compartment even when *shi *function was simultaneously inactivated (Figure 4C' and 4D'). The reduction of cytoplasmic particles of Hh-GFP confirms that endocytosis is inhibited in these experiments (Figure 6, discussed below). These results directly demonstrate that Hh does not require Shi function to move into and across target cells in the anterior compartment. Based on these observations, we conclude that Shi-dependent transcytosis is not essential for movement of HhNp-GFP.

In contrast to the results with constitutively expressed HhNp-GFP which accumulated on the basolateral membranes of the anterior compartment in the absence of Shi [14], induced HhNp-GFP was predominantly found in particles and no basal membrane accumulation was observed (Figure 4). These particles could be detected at both 8 and 24 hours when Shi-dependent endocytosis is blocked (Figure 4C–D) and could be found in both apical and basolateral positions.

The distribution of HhNp-GFP particles in the absence of Shi function was quantified as described above. Overall, the distribution profiles are similar for HhNp-GFP in the wild-type and *shi*^*ts*1 ^mutant backgrounds (Figure 5 and Additional File 4) with the majority of HhNp-GFP found within 8 μm of the A/P boundary (Table 1). While the median values were not statistically significantly different in the *shi*^*ts*1 ^mutant compared to a wild-type background, the percentage of particles within 8 μm as well as the distance of the 90^th ^percentile was significantly different in the *shi*^*ts*1 ^mutant (Table 2). These results suggest that while HhNp-GFP movement through the anterior compartment is not drastically altered when Shi-mediated endocytosis is blocked, Shi function does contribute to the shape of the Hh gradient; following inhibition of Shi, the HhNp-GFP gradient is less steep with a lower percentage of particles retained near the A/P boundary.

The distribution of newly synthesized HhN-GFP following *shi *inactivation was also examined. Under these conditions, HhN-GFP predominantly accumulated at the apical surface of the cells (Figure 4G–H), although some basolateral punctate structures were still detected. HhN-GFP could also be observed in the lumenal space between the peripodial and disc proper cell layers, consistent with previous reports where HhN has been shown to traverse the lumen [14,22]; interestingly, at least some of the lumenal HhN-GFP was present as punctate structures (data not shown). Quantitative analysis of total HhN-GFP particle distribution in these experiments was not possible since the extracellular accumulation in the *shi*^*ts*1 ^mutant prevented reliable identification of individual particles. Nonetheless, visual inspection of the HhN-GFP distribution clearly indicates that Shi-dependent endocytosis is not essential for movement of HhN-GFP across the anterior compartment and that much higher levels of apical HhN-GFP accumulate in the absence of Shi function (Figure 4G–H). These results suggest that most HhN-GFP is apically secreted and then degraded via Shi-dependent endocytosis. However, the presence of some basolateral HhN-GFP in these experiments indicates that not all HhN-GFP is apically secreted.

### Ptc-negative punctate structures require cholesterol and endocytosis

In all of the experiments using inducible Hh-GFP, punctate staining patterns were observed. These particles were classified into four groups using Phalloidin, which labels cortical actin near the cell surface, and an antibody specific for the Hh receptor Ptc (Figure 6, Additional Files 6 and 7). Approximately 60 percent of induced HhNp-GFP or HhN-GFP particles in the anterior compartment of wild type discs were at or near the cell surface and classified as "surface-associated" (Table 3); a substantial fraction of these particles were associated with Ptc. Within the 40 percent of HhNp-GFP that was cytoplasmic, there was an even distribution of HhNp-GFP cytoplasmic vesicles with and without Ptc (Table 4). In contrast, nearly all cytoplasmic HhN-GFP was associated with Ptc, suggesting that the cholesterol modification could mediate Ptc-independent endocytosis (Table 4). However, we cannot exclude the possibility that the Hh vesicles without Ptc could arise from Hh dissociating from Ptc after internalization.

**Table 3:** Hh-GFP co-localization with Ptc in wing discs

	Cytoplasmic			Surface-associated		
	%Total	%Ptc	%no Ptc	%Total	%Ptc	%no Ptc
HhNp-GFP	40 ± 7	22 ± 7	18 ± 2	60 ± 8	16 ± 6	44 ± 7
HhN-GFP	40 ± 12	37 ± 11	3 ± 2	60 ± 12	22 ± 8	38 ± 11
HhNp-GFP(*shi*^*ts*1^)	6 ± 2	0	6 ± 2	94 ± 2	27 ± 1	67 ± 1

3 samples were counted for each genotype. Phalloidin co-localization was counted as surface-associated.

**Table 4:** Non-membrane-associated Hh-GFP co-localization with Ptc in wing discs

	% Co-localized with Ptc	% Not co-localized with Ptc
HhNp-GFP	55 ± 7	45 ± 7
HhN-GFP	92 ± 3	8 ± 3
HhNp-GFP(*shi*^*ts*1^)	0	100

3 discs were counted for each genotype

In *shi*^*ts*1 ^animals, very few cytoplasmic HhNp-GFP particles could be detected (6 percent of total, Table 3) and none of them appeared to co-localize with Ptc (Table 4). This result confirms that inhibition of Shi blocks most endocytosis of Hh, including all Ptc-dependent endocytosis. The number of HhNp-GFP that does not co-localize with Ptc is also reduced. We cannot definitively conclude whether the remaining Ptc-negative Hh-GFP particles classified as cytoplasmic are due to incomplete cell surface labeling with Phallodin or represent cytoplasmic particles formed by a Ptc- and Shi-independent mechanism.

Since inhibition of Shi blocks most or all cytoplasmic particles (Table 3), but leads to greater movement through the anterior compartment (Table 1), our results indicate that transcytosis does not play a major role in spreading HhNp-GFP. In addition, we have observed a potential Ptc-independent mechanism for HhNp-GFP uptake that is dependent on both the cholesterol modification and endocytosis.

## Discussion

### Hh-GFP distribution and gradient formation

We have generated inducible and functional GFP-tagged versions of full length and N-terminal Hh, which allows us to study newly synthesized Hh movement and distribution in live samples, as well as fixed tissues. Our initial analysis of HhNp-GFP localization in living tissue demonstrated similar localization to endogenous HhNp, in particles that were mostly endosomes in the anterior. Upon close examination of HhNp-GFP and HhN-GFP distribution, both were found in punctate structures that localized more apically in the anterior compartment, although basolateral structures were also observed. We have also seen that the Hh gradient appears to require a minimum of 24 hours to fully form. We approximate the minimum HhNp rate of movement to be at least 1 μm/hour (8 μm distance of the 90^th ^percentile over the first 8 hours) and the 90^th ^percentile distance at 12 μm, at the furthest time point. This rate of Hh distribution is a minimum calculated rate, and is slower than the reported rate of Decapentaplegic (Dpp) gradient formation of 6–8 hours [44] and the speed of Activin diffusion of 300 μm in a few hours [45]. However, more time points are needed to determine the exact rate of Hh gradient formation for comparison to diffusion or transcytosis rates.

### HhNp gradient formation through planar diffusion

Previous studies used a block in endocytosis to try to separate the mechanisms of diffusion, which should not require endocytosis, and transcytosis, which should require endocytosis. We expressed wild type Shi in the posterior cells of *shi*^*ts*1 ^mutant discs and studied diffusion versus transcytosis of a pulse of newly produced Hh. This enabled a simultaneous block of endocytosis in the target cells and production of Hh to examine Hh movement. Unexpectedly, we observed HhNp-GFP particles even though we had blocked endocytosis. Upon closer examination, almost all of these particles were associated with the cell surface and not cytoplasmic, indicating these were not intracellular transcytotic vesicles. This suggests that cycles of vesicular endocytosis and exocytosis are unlikely to contribute to movement of HhNp. The absence of HhNp-GFP particles in the lumen when endocytosis was blocked indicates that wild-type Hh has restricted planar movement, as previously demonstrated [14]. Statistical analysis indicated that the median values were not significantly different whereas the further distance of the 90^th ^percentile and the lower percentage of vesicles within 8 μm was statistically significant (Table 2). This indicates that while endocytosis is required for properly shaping the gradient, particularly near the A/P boundary, it is not required for HhNp to move into the anterior compartment. This also suggests that the differences in HhNp-GFP distribution in wild type versus *shi*^*ts*1 ^mutant background are not as dramatic as HhNp-GFP versus HhN-GFP. However, small differences can still have a significant effect on signaling, particularly when local concentrations of HhNp are important in cell fate determination [28,34]. These observations support the model of HhNp-GFP distribution via planar diffusion.

### Cholesterol is required for the steep HhNp gradient

We observed that HhN-GFP was able to travel three times faster than HhNp-GFP (27 μm versus 8 μm distance of the 90^th ^percentile over the first 8 hours) and to target more distant cells in the anterior compartment (31 μm distance of the 90^th ^percentile at the furthest time point), consistent with earlier observations that HhN had a longer range than HhNp [14,19,20]. HhNp-GFP has a steep gradient with a sharp decline and the cholesterol is required for forming this steep gradient, as demonstrated by the higher percentage of HhNp-GFP particles within the first 8 μm from the expressing cells than HhN-GFP. HhNp activates higher levels of the short range target genes *en *and *ptc *[14,20], and this suggests that the purpose of the cholesterol modification is to restrict HhNp closer to the expressing cells, resulting in a precise region of short range target gene activation.

HhN-GFP without the cholesterol was also able to travel into the anterior compartment in the absence of endocytosis. When endocytosis is blocked, HhN-GFP was found to accumulate at high levels in the extracellular lumenal space between the disc proper and the peripodial layer. This observation is similar to a previous report [14], suggesting that HhN-GFP is free to diffuse three-dimensionally, and again evidence that the cholesterol acts to restrict HhNp movement.

Previous studies have observed that HhN is capable of being secreted from the peripodial cells and signals to the columnar epithelial cells [14,22]. However, we believe that peripodial HhN-GFP has a minimal contribution on the distribution profile. Since the posterior compartment of the peripodial membrane overlies the section that the measurement data originated from, we attempted to take the contribution from the peripodial membrane into account by subtracting the HhN signal from the end of the distribution profile from the rest of the data set. Subtracting this signal did not significantly alter our results and conclusions.

### Ptc-negative vesicles

Further examination of the Hh-GFP particles detected four classes of particles including Ptc-negative cytoplasmic vesicles. Interestingly, we observed that HhNp-GFP has a significantly higher percentage of these Ptc-negative particles than HhN-GFP. Previous studies have also observed intracellular Ptc-negative Hh vesicles and shown that they are still present in cells mutant for *ptc *[14,34,35,37]. However, there are distinct differences in our observations from previous studies. Previous results showed that most of HhNp co-localizes with Ptc internally [14,34] while a high proportion of HhN does not co-localize with Ptc [14]. Since these studies were done at the Hh gradient steady state, the Ptc receptor could have been saturated by this point, resulting in higher levels of HhNp and Ptc co-localization [14,34]. Additionally, clones looking at HhN and Ptc co-localization appear to be outside of the high Ptc-expressing stripe [14] and since there is less Ptc there, one might conclude that there is less Ptc co-localization.

The Ptc-negative vesicles could represent HhNp-GFP that has somehow dissociated from Ptc after internalization, possibly as a part of a recycling mechanism. *C. elegans *Hh-related peptides are sorted to multivesicular bodies (MVBs), then recycled back to the apical surfaces for secretion [46]. However, a recent study reported that Hh does not go through the Rab11-mediated recycling pathway [22]. Another possibility is the presence of another receptor besides Ptc, such as the low density lipoprotein receptor, Megalin, previously demonstrated to interact with vertebrate Shh [47]. The absence of these vesicles when endocytosis is blocked indicates that they are not essential for transport although further studies of these Ptc-negative vesicles are required to elucidate the nature of these vesicles.

## Conclusion

Previous publications have reported that cholesterol modification of Hh is important for its distribution by analyzing target gene expression. However, these studies showed discrepancies in the range of non-cholesterol modified HhN and in the apicobasal localization of the different forms of Hh leading to different models of Hh distribution. We have developed a system to induce a pulse of newly synthesized Hh that can be used to further characterize formation of the Hh gradient. Inducible expression, where a pulse of newly synthesized protein is generated, would enable observations of movement during gradient formation instead of at the gradient steady-state, and at protein concentrations closer to endogenous levels. Since contradictory observations exist about the mechanisms regulating morphogen distribution and gradient formation not only for Hh but also for Dpp and Wingless (Wg), clarification could come from using an inducible system together with quantitative measurements.

We have observed that HhN can be detected at a longer range than modified HhNp. We have quantitatively demonstrated that newly synthesized HhNp-GFP distribution requires cholesterol and can occur without endocytosis in agreement with published results. Additionally, HhNp-GFP is detected in intracellular vesicles that do not co-localize with Ptc but we conclude that they are not essential for Hh distribution since they are not observed in endocytosis defective cells. Furthermore, in our inducible system, the modified and unmodified forms of Hh localize at both apical and basolateral regions suggesting there may not be a preferential region for movement. Our data support a model where the cholesterol modification of Hh is required to restrict its planar diffusion, thereby forming a steep gradient.

## Methods

### Drosophila stocks and genetic experiments

The following mutants and transgenes have been previously described: *hh*^*GS*1^, an amorphic allele also known as *hh*^11 ^[48]; *shi*^*ts*1 ^[49], UAS-*shi*^+ ^also known as UAS-*dynamin *[44], *tubulin-Gal80ts*^2 ^[43], UAS-*dsRed *[50], UAS-*GFP-dally-like *[28], UAS-*ptc-YFP *[51], *Hh-Gal4 *[40], *Ptc-Gal4 *[52], and *71B-Gal4 *[38].

UAS-*hhF-GFP *and UAS-*hhN-GFP *transgenic flies were generated. These fusion proteins are similar to those previously described [34,37]. To construct UAS-*hhF-GFP*, *GFP *was inserted in frame into full length *hh *between amino acids 254 (H) and 255 (V). The PCR primers used are as follows: GAGTCGCGGCCGCATCATGGATA and ATGGATCCGTGGGAACTGATCGACGAATC for the first half of full length *hh *(*hh1*); ACGGATCCATGGTGSGCAAGGGCGAG and ACGAATTCCTTGGTACAGCTCGTCATGCC for *GFP*; AGGAATTCGTGCACGGCTGCTTCAC and TGGGTACCCAGGATTCCATCATCAAT for the second half of full length *hh *(*hh2*). PCR fragments were generated using *hh *cDNA and eGFP-N1 (Clontech) plasmids as templates, and cloned into pBluescript (Stratagene) in the following restriction enzymes sites (underlined in PCR primer sequence): NotI/BamHI for *hh1*, BamHI/EcoRI for *GFP*, and EcoRI/KpnI for *hh2*. The full *hhF-GFP *(*hh1-GFP-hh2*) sequence was then cloned into pUASp2 [53] using the NotI/KpnI restriction enzyme sites. To construct UAS-*HhN-GFP *that lacks the cholesterol modification, *hh *was truncated at amino acid 257 (G) and *GFP *was cloned in frame immediately behind truncated *hh*. The PCR primers used were: GAGGTACCGAGAAACAGCAAACAACGAGTCTTAG and ATGGATCCAAGCCGTGGGAACT for *hhN*. The HhN PCR fragment was cloned into pUASp that already contained *GFP *using KpnI/BamHI restriction enzyme sites (underlined in PCR primer sequence). All PCR fragments were sequenced (Macrogen). Each construct was co-injected with the delta 2–3 transposase helper plasmid into w^1118 ^embryos to generate transgenic lines.

For rescue and localization experiments, the following larval genotypes were used:

*En-Gal4*; UAS-*hhF-GFP hh*^*GS*1^

UAS-*dsRed*/+; UAS-*hhF-GFP*/*Hh-Gal4*

UAS-*hhF-GFP*/*71B-Gal4*

UAS-*hhF*/*71B-Gal4*

UAS-*CD8-GFP*/+; *71B-Gal4*/+

UAS-*myrpalm-CFP*; UAS-*hhF-GFP*/*Hh-Gal4*

UAS-*ptc-YFP*/*Ptc-Gal4*

*Ptc-Gal4*/+; UAS-*GFP-Dlp*

For experiments analyzing Hh temporal distribution, the following larval genotypes were generated:

Hsflp UAS-*dsRed*/+; UAS-*hhF-GFP hh*^*GS*1^/*Hh-Gal4 tub-Gal80ts*^2^

Hsflp UAS-*dsRed*/+; UAS-*hhN-GFP hh*^*GS*1^/*Hh-Gal4 tub-Gal80ts*^2^

To examine Hh distribution in discs with an endocytosis-defect in the anterior compartment, a *shi *mutant allele was used. Shi is the *Drosophila *homologue of mammalian GTPase Dynamin and the *shi*^*ts*1 ^mutant allele is a temperature sensitive allele with the permissive temperature at 18°C and the restrictive temperature at 32°C. These larvae have a *shi *mutant background at the restrictive temperature which coincides with the expression of wild-type Shi under the Gal80-Gal4 system to rescue the mutant phenotype in the posterior compartment. For these experiments, the following larval genotypes were generated:

*shi*^*ts*1 ^FRT19A; UAS-*shi*^+^/Hsflp UAS-*dsRed*; UAS-*hhF-GFP hh*^*GS*1^/*Hh-Gal4 tub-Gal80ts*^2^

*shi*^*ts*1 ^FRT19A: UAS-*shi*^+^/Hsflp UAS-*dsRed*; UAS-*hhN-GFP hh*^*GS*1^/*Hh-Gal4 tub-Gal80ts*^2^

Induction of Hh-GFP expression using Gal80ts:

Larvae were raised at 18°C. Third instar larvae (day 10–15 at the Gal80 permissive temperature 18°C) were reared at 32°C (the Gal80 restrictive temperature) for 8, 24, or 72 hours. Larvae were dissected at room temperature, and fixed immediately before immunostaining (total time between removal from permissive temperature to fixation was 5–10 minutes).

### Imaginal Disc Preparation and Immunostaining

Larvae were removed from 32°C to room temperature, dissected, and fixed immediately (total time of 5–10 minutes). Immunostaining was performed according to Patel [54]. For induction studies, the following modifications were used. Briefly, discs were fixed in 4% paraformaldehyde in PBS for 20 minutes, washed in PBS, blocked for 30 minutes in PBS with 0.5% BSA and 5% normal goat serum (NGS), incubated in primary antibody for 1 hour in PBS with 0.1% TritonX-100, 0.5% BSA, and 5% NGS, washed in PBS for 20 minutes, incubated in secondary antibody diluted in the initial blocking solution for 30 minutes, and washed for 30 minutes. Discs were mounted in 50% glycerol/PBS. Strips of double-stick tape were added to the slides as spacers to prevent compression of the discs.

Primary antibodies were used at the following concentrations: rat anti-DCAD 1:50 [55]; mouse anti-Ptc 1:50 [56], rabbit anti-dsRed 1:500 (Clontech), rat anti-Ci 1:10 [57], mouse anti-GFP 1:250 (Molecular Probes). Secondary antibodies used were anti-rabbit and anti-rat Alexa 555 1:1000, anti-mouse Alexa 647 1:1000 (Molecular Probes), and anti-rabbit Cy3 1:600 (Jackson Laboratories).

Protocols for membrane labeling, endosome labeling, and extracellular labeling have been described previously [41,44,58]. Briefly, to label membranes, discs were mounted in 9 μM FM4-64 (Molecular Probes) diluted in 1 × PBS and incubated for 20 minutes at 25°C before live imaging. To label endocytic compartments, discs were incubated with 17 μM tetramethylrhodamine-dextran (3000 MW, Molecular Probes) diluted in incomplete M3 media for 10 minutes in the dark at 25°C, washed, and mounted in incomplete M3 media, and incubated for 30 minutes at 25°C before live imaging. For extracellular labeling, discs were incubated with anti-GFP (1:250 dilution in incomplete M3 media) for 30 minutes on ice before being washed 5× with ice cold 1 × PBS and fixed in 4% paraformaldehyde/PBS for 20 minutes at room temperature. Subsequent processing is the same as stated above for immunostaining. For Alexa 546 and Alexa 647 Phalloidin (Molecular Probes) labeling, Phalloidin was diluted 1:40 in blocking solution and added during secondary antibody incubation step for 20–30 minutes before washes and mounting.

### Microscopy, Image Acquisition, and Analysis

Fluorescence images were collected on a Leica TCSSP2 AOBS confocal microscope, and processed using the Leica Confocal Software 2.5 Build 1347, Adobe Photoshop 7.0, AutoDeBlur & AutoVisualize X 1.4.1 (MediaCybernetics) and Imaris 5.0.1 (Bitplane).

To count Hh-GFP containing vesicles, 79.35 um^2 ^XY sections were collected using the 63 × objective, in the center of the wing pouch every 0.5 μm for the entire depth of the disc (60–100 μm which was approximately 120–200 sections). All discs were imaged under identical microscope settings for laser power, pinhole, and gain. Quantitative analysis was done in the Imaris software program.

### Vesicle identification and distance measurement

For negative controls (the 0 hour time point), surface intensity thresholds were set just below background levels. This resulted in some background to be incorrectly identified as real signal. After this step, surfaces were sorted according to volume and any surface with a volume of less than 0.03 um^3 ^was discarded, leaving only a few background surfaces. This strategy was used to maximize true Hh-GFP signal identification and minimize incorrect identification of background signal.

We applied this strategy to Hh-GFP expressing samples. Surface intensity thresholds were set to just below background, then sorted by volume, and surfaces with volumes more than 0.03 μm^3 ^were counted and measured.

After vesicle identification, a "Distance Transformation" tool generated a distance map from the UAS-dsRed signal marking the expressing cells. This map was applied to the surfaces to determine the shortest distance of vesicles from the Hh expressing cells. The distance measurements were then imported into Excel and plotted to generate distribution profiles. For each sample, total particle numbers were normalized by dividing the number of particles at each distance by the total number of particles for that sample. The normalized data was then averaged to generate the overall distribution profile.

### Statistical Analysis

To determine whether the measurement values of median, 90^th ^percentile distance, and percentage at 8 μm were significant between genotypes and/or time points, the measurements were subjected to the analysis of variance (ANOVA). The natural log of the medians, natural log of the 90^th ^percentile distances and the raw values for percentage at 8 μm were analyzed as these met the normality assumptions of the ANOVAs. Specifically, the Tukey's HSD test was used to determine significance. There was no significant interaction between the time factor and the genotype factor. Therefore, the significance of the main effects (time irrespective of genotype or genotype irrespective of time) are described. P-values less than 0.05 are considered significant.

### Quantification of Ptc and Phalloidin co-localization

Particles were identified in the same way as for distance measurement quantification. After particle identification, each particle was analyzed through the z-stack for co-localization with Ptc and Phalloidin, then sorted into the appropriate category.

### Western Blot

Using the *71B-Gal4 *drivers to express various transgenes, salivary glands were dissected from the following larvae: *w*^1118 ^(10 glands), UAS-*CD8:GFP *(5 glands), UAS-*hhN-GFP hh*^*GS*1 ^(10 glands), UAS-*hhF-GFP hh*^*GS*1 ^(10 glands), and UAS-*hhF *(15 glands) and put on ice. Salivary glands were put in 40 μL of sample buffer and broken up with a Dounce Homogenizer. The lysate was spun down and the supernatant was collected and loaded on a 10% polyacrylamide gel for SDS-PAGE. The blot was first labeled for presence of GFP, then stripped and re-probed for a tubulin loading control. Antibodies used were rabbit anti-GFP 1:1500 (Molecular Probes), mouse anti-tubulin 1:3000 (Oncogene), and anti-rabbit and anti-mouse HRP 1:20000 (Jackson Laboratories).

### Wing Preparations

Wings were collected from adult flies expressing various transgenes under the control of the *71B-Gal4 *driver. Whole flies were put in isopropanol, wings were pulled off fly bodies and mounted in 50% Canada Balsam/isopropanol.

## Authors' contributions

VFS generated the constructs, carried out the genetics, immunofluorescence and localization studies, quantitative analysis, and drafted the manuscript. KAJ performed the Western blot analysis and generated constructs. MB participated in experimental design, coordination, and writing of the manuscript. IT conceived the study, participated in its design, coordination, and writing of the manuscript.

## Supplementary Material

Additional File 1 Extracellular Hh localizes apically in particles and basolaterally in particles and along the membrane. (A-B) Ptc-YFP (green, A) and GFP-Dlp (green, B), and extracellular labeling (red). As controls for the extracellular labeling protocol with the anti-GFP antibody, Ptc-YFP was used as a negative control since YFP is attached to the cytoplasmic region of Ptc and GFP-Dlp was used as a positive control since GFP is attached to the extracellular region of Dlp. (C-D) HhNp-GFP (green), extracellular labeling with anti-GFP (red), and DCAD to mark the apical region (blue- C, C'', D, D''; purple-C''', D'''). Two separate examples of extracellular HhNp-GFP, extracellular HhNp-GFP is detected in the anterior apically in particles (arrows in C and D), and basolaterally both in particles (arrowheads in C and D) and with a membrane association (bracket in C and D). Click here for file

Additional File 2 Hh gradient forms by 24 hr of induction. (A-D) Induced expression of HhNp-GFP (A-B) and HhN-GFP (C-D) in wild-type background. (A-D) 25 μm projections; (A'-D') 20 μm Z-section projections. 24 and 72 hr distribution of HhNp-GFP appears similar, also seen for HhN-GFP. Scale bar: 5 μm Click here for file

Additional File 3 Individual histograms of raw data with median, 90^th ^percentile distance and % within 8 μm values. (A) HhNp-GFP at 8 hr time point: n = 5. (B) HhNp-GFP at 24 hr time point: n = 16. (C) HhNp-GFP at 72 hr time point: n = 5. (D) HhN-GFP at 8 hr time point: n = 4. (E) HhN-GFP at 24 hr time point: n = 7. (F) HhN-GFP at 72 hr time point: n = 6. (G) HhNp-GFP in *shi*^*ts*1 ^mutant background at 8 hr time point: n = 4. (H) HhNp-GFP in *shi*^*ts*1 ^mutant background at 24 hr time point: n = 7. Click here for file

Additional File 4 Full distribution profiles of Hh-GFP. (A-C) Mean of normalized HhNp-GFP (green) versus HhN-GFP (red) distribution profiles in a wild-type background at 8 (A), 24 (B), and 72 hr (C) time points. All samples were normalized to generate percentages of particles at the distances. Normalized data was then averaged to generate distribution profiles. More HhNp-GFP is found closer to the A/P boundary (0 on the x-axis) than HhN-GFP at 8 hr (A), 24 hr (B), and 72 hr (C) time points. (D-E) Mean of normalized HhNp-GFP distribution profiles in wild-type background (green) versus *shi*^*ts*1 ^mutant background (blue). More HhNp-GFP is also found closer to the A/P boundary (0 on the x-axis) in the wild-type background than in the *shi*^*ts*1 ^mutant background at 8 (D) and 24 hr (E). The same HhNp-GFP distribution profiles in the wild-type background from A and B are used for D and E, respectively. Click here for file

Additional File 5 Constitutively expressed HhNp-GFP accumulates at basal membranes after blocking endocytosis. (A-D) HhNp-GFP (green) localization prior to (A) and after an 8 hr (B-D) endocytosis block in the *shi*^*ts*1 ^mutant background with Phalloidin (red) as a cell surface marker; 3 μm Z-section projections. HhNp-GFP does not normally accumulate at cell surfaces in the anterior compartment (A/P boundary is marked by a solid white line). At the *shi*^*ts*1 ^permissive temperature, HhNp-GFP accumulates primarily at the basal cell surfaces in the anterior to varying degrees (B-high, C-intermediate, D-low). Scale bar: 5 μm Click here for file

Additional File 6 Quantification scheme of Hh-GFP membrane localization and co-localization with Ptc. (A) Hh-GFP surfaces were generated to identify particles based on the same criteria used in particle distance measurements. Each particle was individually located for particle classification (white arrow connected to box). (B-D) Classification of particles. After particle identification, Hh-GFP particles (green) were located in XY, XZ, and YZ views (B). Co-localization was determined with Phalloidin (purple, C) and Ptc (red, D) in these views through the z-stack (white arrows identify the same particle in XZ and YZ views that was originally identified in the XY view). Scale bar: 5 μm Click here for file

Additional File 7 Non-Ptc containing Hh-GFP particles require cholesterol but not endocytosis. (A-C) Z-section of Ptc co-localization with HhNp-GFP (A), HhN-GFP (B), and HhNp-GFP in the *shi*^*ts*1 ^background (C) after expression induced for 8 hr. (A-C) Hh-GFP (green) labeled with Phalloidin (purple). (A'-C') Hh-GFP (green) labeled with Ptc (red). (A''-C'') Hh-GFP only. (A'''-C''') Ptc only. 4 classes of HhNp-GFP particles are seen: non-Phalloidin associated (cytoplasmic) with Ptc (white arrow), non-Phalloidin associated (cytoplasmic) without Ptc (white arrowhead), Phalloidin (membrane) associated with Ptc (yellow arrow), Phalloidin (membrane) associated without Ptc (yellow arrowhead). Most HhNp-GFP particles are Phalloidin-associated and do not contain Ptc, but cytoplasmic particles have a relatively even distribution with and without Ptc. More HhN-GFP also localizes with Phalloidin, and almost all of the cytoplasmic HhN-GFP particles contain Ptc. HhNp-GFP particles in *shi*^*ts*1 ^mutant background are Phalloidin-associated and many do not contain Ptc. The A/P boundary is marked by a solid white line. Scale bar: 5 μm Click here for file
