# Supplementary figures and images for: Quantitative analysis of Hedgehog gradient formation using an inducible expression system
Source: BMC Dev Biol. 2007 May 7;7:43. doi: 10.1186/1471-213X-7-43 (PMC1885436; doi:10.1186/1471-213X-7-43)

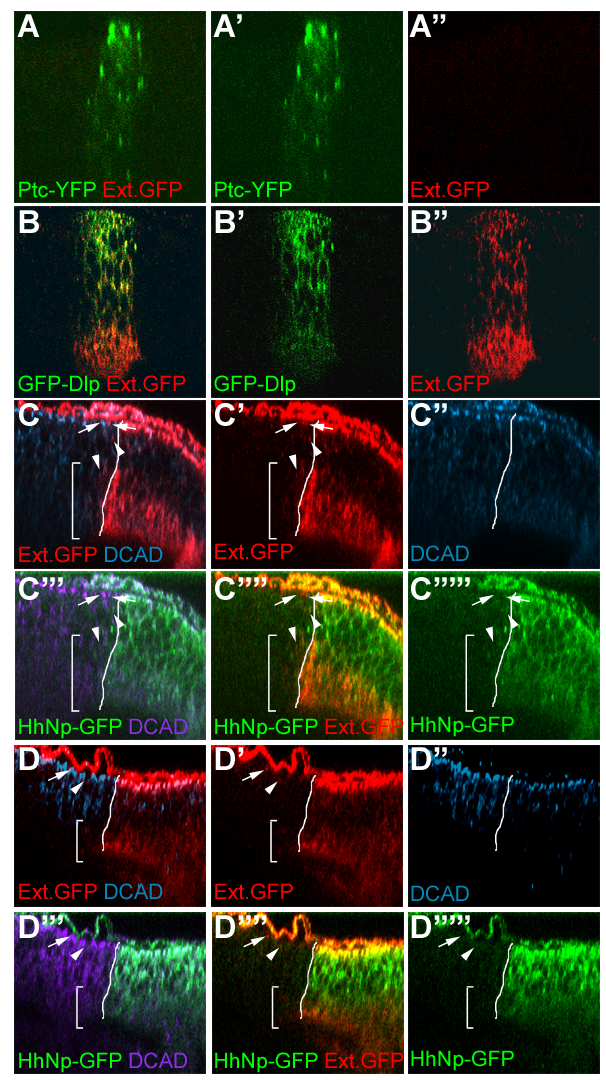

Supplement: Additional File 1 — Extracellular Hh localizes apically in particles and basolaterally in particles and along the membrane. (A-B) Ptc-YFP (green, A) and GFP-Dlp (green, B), and extracellular labeling (red). As controls for the extracellular labeling protocol with the anti-GFP antibody, Ptc-YFP was used as a negative control since YFP is attached to the cytoplasmic region of Ptc and GFP-Dlp was used as a positive control since GFP is attached to the extracellular region of Dlp. (C-D) HhNp-GFP (green), extracellular labeling with anti-GFP (red), and DCAD to mark the apical region (blue- C, C'', D, D''; purple-C''', D'''). Two separate examples of extracellular HhNp-GFP, extracellular HhNp-GFP is detected in the anterior apically in particles (arrows in C and D), and basolaterally both in particles (arrowheads in C and D) and with a membrane association (bracket in C and D). [file 1471-213X-7-43-S1.tiff]

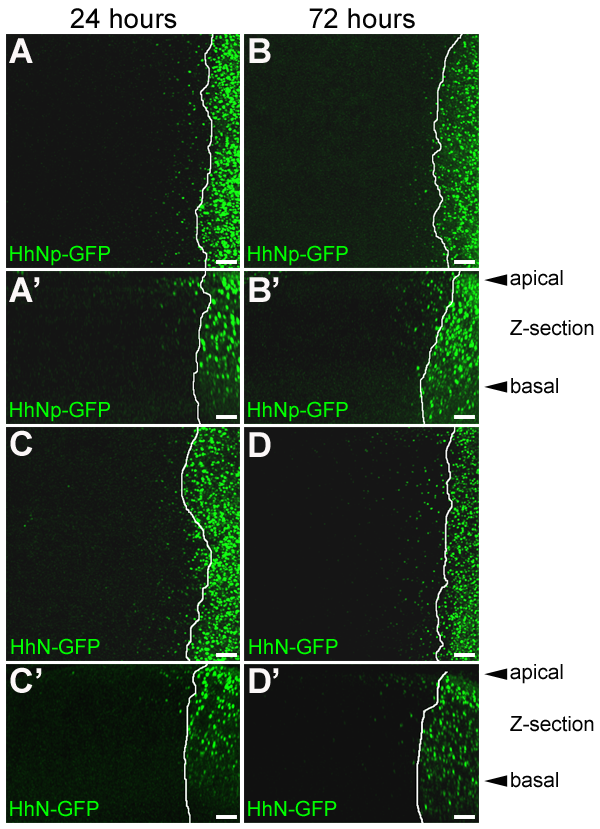

Supplement: Additional File 2 — Hh gradient forms by 24 hr of induction. (A-D) Induced expression of HhNp-GFP (A-B) and HhN-GFP (C-D) in wild-type background. (A-D) 25 μm projections; (A'-D') 20 μm Z-section projections. 24 and 72 hr distribution of HhNp-GFP appears similar, also seen for HhN-GFP. Scale bar: 5 μm [file 1471-213X-7-43-S2.tiff]

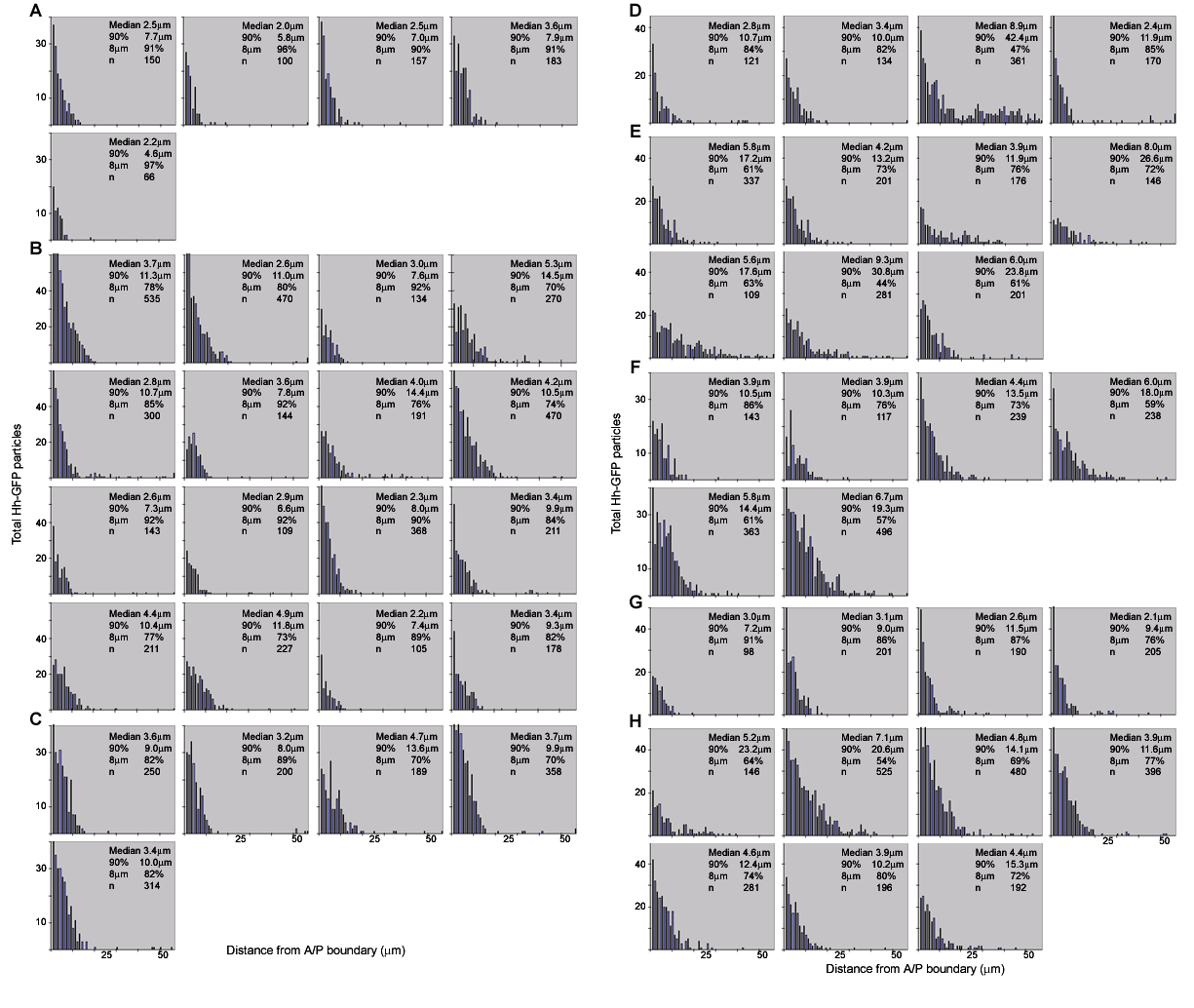

Supplement: Additional File 3 — Individual histograms of raw data with median, 90th percentile distance and % within 8 μm values. (A) HhNp-GFP at 8 hr time point: n = 5. (B) HhNp-GFP at 24 hr time point: n = 16. (C) HhNp-GFP at 72 hr time point: n = 5. (D) HhN-GFP at 8 hr time point: n = 4. (E) HhN-GFP at 24 hr time point: n = 7. (F) HhN-GFP at 72 hr time point: n = 6. (G) HhNp-GFP in shits1 mutant background at 8 hr time point: n = 4. (H) HhNp-GFP in shits1 mutant background at 24 hr time point: n = 7. [file 1471-213X-7-43-S3.tiff]

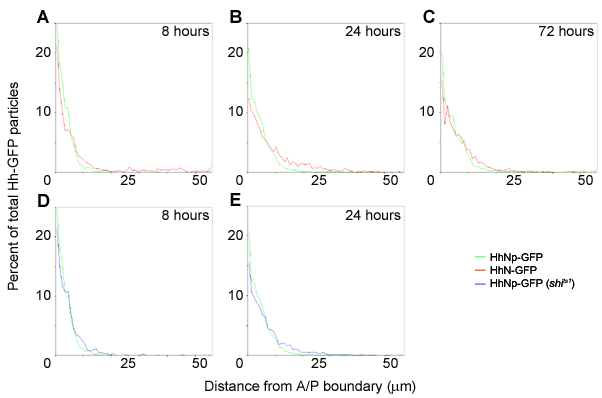

Supplement: Additional File 4 — Full distribution profiles of Hh-GFP. (A-C) Mean of normalized HhNp-GFP (green) versus HhN-GFP (red) distribution profiles in a wild-type background at 8 (A), 24 (B), and 72 hr (C) time points. All samples were normalized to generate percentages of particles at the distances. Normalized data was then averaged to generate distribution profiles. More HhNp-GFP is found closer to the A/P boundary (0 on the x-axis) than HhN-GFP at 8 hr (A), 24 hr (B), and 72 hr (C) time points. (D-E) Mean of normalized HhNp-GFP distribution profiles in wild-type background (green) versus shits1 mutant background (blue). More HhNp-GFP is also found closer to the A/P boundary (0 on the x-axis) in the wild-type background than in the shits1 mutant background at 8 (D) and 24 hr (E). The same HhNp-GFP distribution profiles in the wild-type background from A and B are used for D and E, respectively. [file 1471-213X-7-43-S4.tiff]

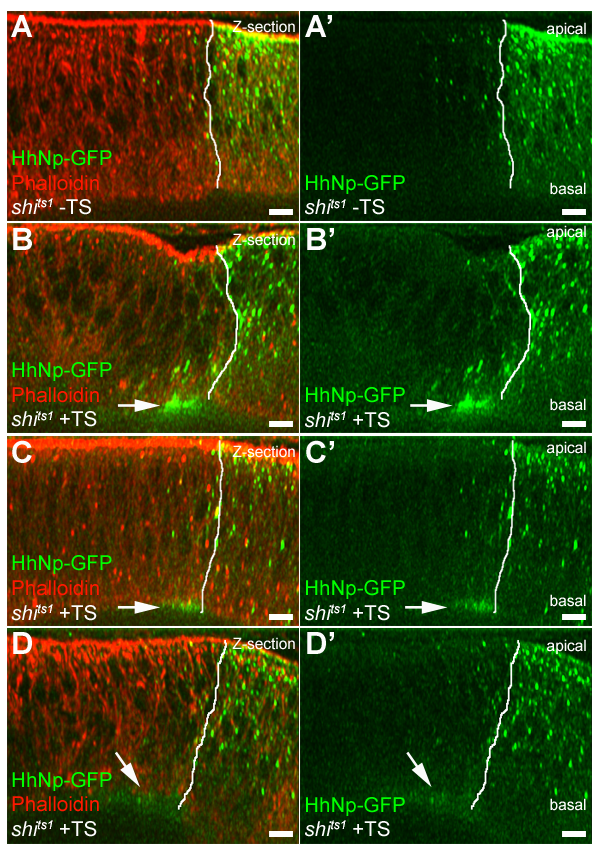

Supplement: Additional File 5 — Constitutively expressed HhNp-GFP accumulates at basal membranes after blocking endocytosis. (A-D) HhNp-GFP (green) localization prior to (A) and after an 8 hr (B-D) endocytosis block in the shits1 mutant background with Phalloidin (red) as a cell surface marker; 3 μm Z-section projections. HhNp-GFP does not normally accumulate at cell surfaces in the anterior compartment (A/P boundary is marked by a solid white line). At the shits1 permissive temperature, HhNp-GFP accumulates primarily at the basal cell surfaces in the anterior to varying degrees (B-high, C-intermediate, D-low). Scale bar: 5 μm [file 1471-213X-7-43-S5.tiff]

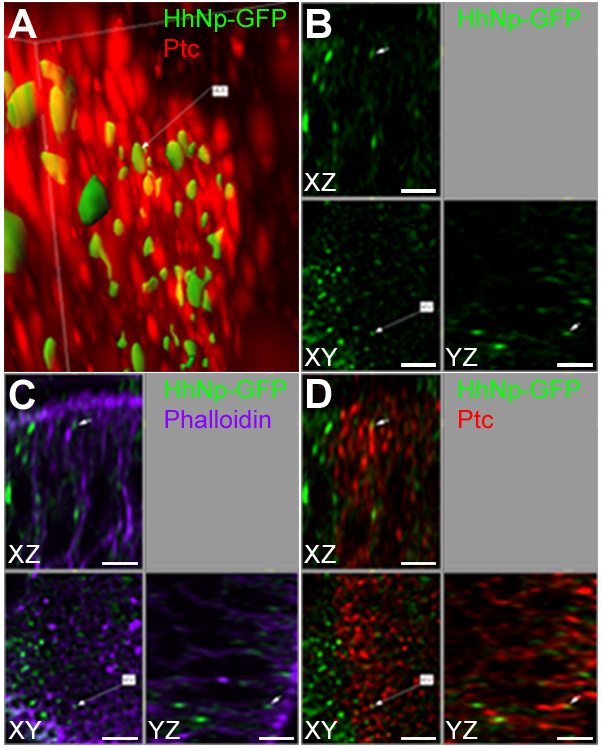

Supplement: Additional File 6 — Quantification scheme of Hh-GFP membrane localization and co-localization with Ptc. (A) Hh-GFP surfaces were generated to identify particles based on the same criteria used in particle distance measurements. Each particle was individually located for particle classification (white arrow connected to box). (B-D) Classification of particles. After particle identification, Hh-GFP particles (green) were located in XY, XZ, and YZ views (B). Co-localization was determined with Phalloidin (purple, C) and Ptc (red, D) in these views through the z-stack (white arrows identify the same particle in XZ and YZ views that was originally identified in the XY view). Scale bar: 5 μm [file 1471-213X-7-43-S6.tiff]

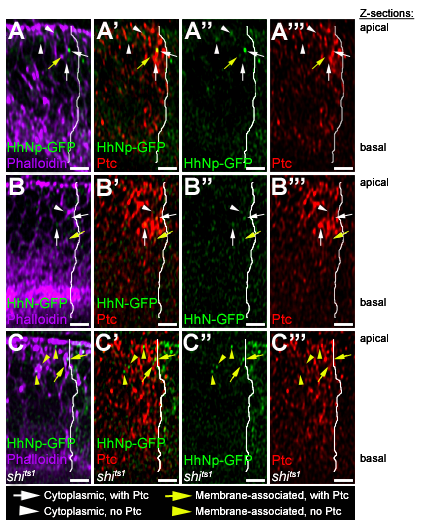

Supplement: Additional File 7 — Non-Ptc containing Hh-GFP particles require cholesterol but not endocytosis. (A-C) Z-section of Ptc co-localization with HhNp-GFP (A), HhN-GFP (B), and HhNp-GFP in the shits1 background (C) after expression induced for 8 hr. (A-C) Hh-GFP (green) labeled with Phalloidin (purple). (A'-C') Hh-GFP (green) labeled with Ptc (red). (A''-C'') Hh-GFP only. (A'''-C''') Ptc only. 4 classes of HhNp-GFP particles are seen: non-Phalloidin associated (cytoplasmic) with Ptc (white arrow), non-Phalloidin associated (cytoplasmic) without Ptc (white arrowhead), Phalloidin (membrane) associated with Ptc (yellow arrow), Phalloidin (membrane) associated without Ptc (yellow arrowhead). Most HhNp-GFP particles are Phalloidin-associated and do not contain Ptc, but cytoplasmic particles have a relatively even distribution with and without Ptc. More HhN-GFP also localizes with Phalloidin, and almost all of the cytoplasmic HhN-GFP particles contain Ptc. HhNp-GFP particles in shits1 mutant background are Phalloidin-associated and many do not contain Ptc. The A/P boundary is marked by a solid white line. Scale bar: 5 μm [file 1471-213X-7-43-S7.tiff]
